# Coping styles in refugees with PTSD: Results from a randomized trial comparing EMDR therapy and stabilization

**DOI:** 10.1371/journal.pone.0310093

**Published:** 2024-09-16

**Authors:** Henriëtte van Heemstra, Niels van der Aa, Trudy Mooren, Daniel Medema, Gerko Vink, Jeroen Knipscheer, Ali Moradi, Rolf Kleber, Jackie June ter Heide

**Affiliations:** 1 ARQ Centrum’45, Diemen, The Netherlands; 2 Department of Clinical Psychology, Utrecht University, Utrecht, The Netherlands; 3 Jellinek, Utrecht, The Netherlands; 4 Team Statushouders, Mentrum, Amsterdam, The Netherlands; Almoosa College of Health Sciences, SAUDI ARABIA

## Abstract

**Background:**

While treatment of posttraumatic stress disorder (PTSD) in refugees is generally effective, many refugees remain symptomatic after treatment. Coping styles could be relevant to PTSD treatment response and as such may be a potential focus of PTSD treatment.

**Aims:**

The study aims to examine 1) if baseline coping styles are related to treatment response after EMDR therapy or stabilization, and 2) if coping styles change during these treatments.

**Method:**

Seventy-two refugees with PTSD were randomly allocated to 12 hours of EMDR therapy or stabilization. A coping questionnaire (COPE-easy) and clinical interview for PTSD (CAPS-IV) were administered before and after treatment and at three-month follow-up. The association between baseline coping styles and PTSD symptom change was examined using regression analysis and a t-test. Changes in coping styles were analyzed using mixed design ANOVA.

**Results:**

No significant relations between baseline coping style levels and PTSD symptom changes were found. Additionally, coping style levels did not change significantly after either treatment.

**Conclusion:**

Contrary to the hypothesis, we did not find any evidence that treatment was related to (changes in) coping style. Addressing pre-treatment coping styles among refugees receiving short-term therapy, may not be required for reducing PTSD. Changing coping styles may need a longer or different type of treatment.

## Introduction

War, violence, persecution and human rights abuses force many people to flee their homes and find refuge in neighboring or other countries. Due to armed conflict, numbers of forcibly displaced people have steadily increased over the past decade and have recently surpassed 80 million [1]. The psychological wellbeing of refugees is threatened not only by traumatic experiences [2, 3] but also by daily stressors in the country of refuge [4]. Consequently, refugees are at higher risk of developing mental health problems than nonrefugees and migrant populations [5–7], with posttraumatic stress disorder (PTSD; 31%) and major depressive disorder (32%) being especially common [8].

Psychological interventions are effective in mitigating PTSD and depression in refugees [9], but unfortunately many remain symptomatic after treatment [10]. We previously reported on the primary outcomes of a randomized controlled trial (RCT) comparing the safety and efficacy of EMDR therapy versus stabilization in refugees with PTSD [11]. In this trial, EMDR therapy was found to be equally safe and efficacious in reducing PTSD symptom severity as stabilization, but the effect sizes were small [11]. An increased understanding of the mechanisms that contribute to psychological recovery in this group, and how these can be promoted, can extend and optimize current treatment options [12].

For traumatized refugees, coping is a relevant mechanism for mental health improvement. Coping can be defined as the set of cognitive and behavioral strategies that people employ to deal with stressful situations [13]. Coping is classified into different styles, including problem-focused coping (acting to solve or improve the situation), emotion-focused coping (focusing on thoughts and feelings related to the situation), support-seeking (seeking practical or mental support to deal with the situation) and avoidant coping (avoiding confrontations with the situation) [14]. For refugees coping is especially relevant since it might impact two important sources of burden often reported for this population. First, coping helps refugees deal with the daily stressors they face [15, 16]. Second, coping is associated with PTSD symptoms among refugees [17]. Previous research [14], reported a cross-sectional association between problem-focused coping and lower PTSD symptoms as well as avoidant coping and higher PTSD symptoms, but no relations between emotion-focused or support-seeking coping and PTSD symptoms were found.

The associations between coping styles and PTSD treatment response have been established, but also seem to vary between samples [18]. For example, pre-treatment avoidant coping predicted higher post-treatment PTSD symptomology among military veterans [19], but had an opposite effect among rape survivors [20]. Following the forementioned studies, the impact of coping styles on PTSD treatment response may be population-specific. For refugees, the associations between coping styles and treatment response are not empirically substantiated and require further examination.

Furthermore, the previously described associations between coping styles and PTSD symptomatology [14] raise the question if coping styles can be improved. While the possibilities of modifying coping among refugees are unknown, helping patients to use beneficial coping styles is defined as a frequent factor in different PTSD treatments [21]. It is relevant to consider the potential impact from treatment on coping from a dual perspective since PTSD therapies can be roughly divided into trauma-focused and non-trauma-focused approaches [22]. EMDR therapy is a trauma-focused treatment in which the reprocessing of traumatic memories is the mechanism of change [23]. Apart from an active overcoming of avoidant coping, change in coping styles is not explicitly addressed in EMDR therapy. Stabilization, on the other hand, is a non-trauma-focused supportive treatment that, among other things, explicitly focuses on fostering adaptive coping styles through various techniques [11, 24]. Based on these differences, an exploration of the associations of the two treatments with coping style is warranted to gain knowledge on the potential changes in coping after PTSD treatment. Consequently, the current study addresses the following questions:

1. Are baseline coping styles related to PTSD treatment response among refugees?
2. Do coping styles change among refugees, during treatment, and do these changes differ between treatment conditions?

As for the first question, based on relevant literature [14] we had the following hypotheses: (1) higher baseline avoidant coping is related to lower post-treatment PTSD reductions and more non-response; (2) higher baseline problem-focused coping is related to more PTSD symptom change and treatment response, (3) baseline support-seeking and emotion-focused coping are not associated with PTSD symptom change or treatment response.

As for the second question, we hypothesized: (4) coping styles change after receiving treatment. Based on the content of the therapies (EMDR therapy aims to tackle the avoidance of trauma-related memories whereas stabilization techniques focus on increasing functional coping) we expected (5) larger reductions for avoidant coping in the EMDR condition, and (6) larger increases for problem-focused, support-seeking and emotion-focused coping styles in the stabilization condition.

## Methods

### Design

Data were used from a randomized controlled trial (RCT) with two arms: EMDR therapy versus stabilization, using blocked simple randomization [11]. Randomization was performed by a researcher who was not involved in the inclusion process. Flipping a coin was used for the random sequence allocation of participants to the different conditions (heads for EMDR, tails for stabilization). PTSD symptom severity and coping style levels were assessed at pre-treatment (T1), post-treatment (T2) and three months follow-up (T3). The trial was approved by the medical-ethics committee of the University of Leiden. Trial registration: NARCIS (Dutch National Academic Research and Collaborations Information System) OND1324839; ISRCTN20310201. For the flowchart and harm definition see the reference of the main publication of this trial [11]. The CONSORT reporting guidelines were used in the preparation of this manuscript [25].

**Fig 1:**
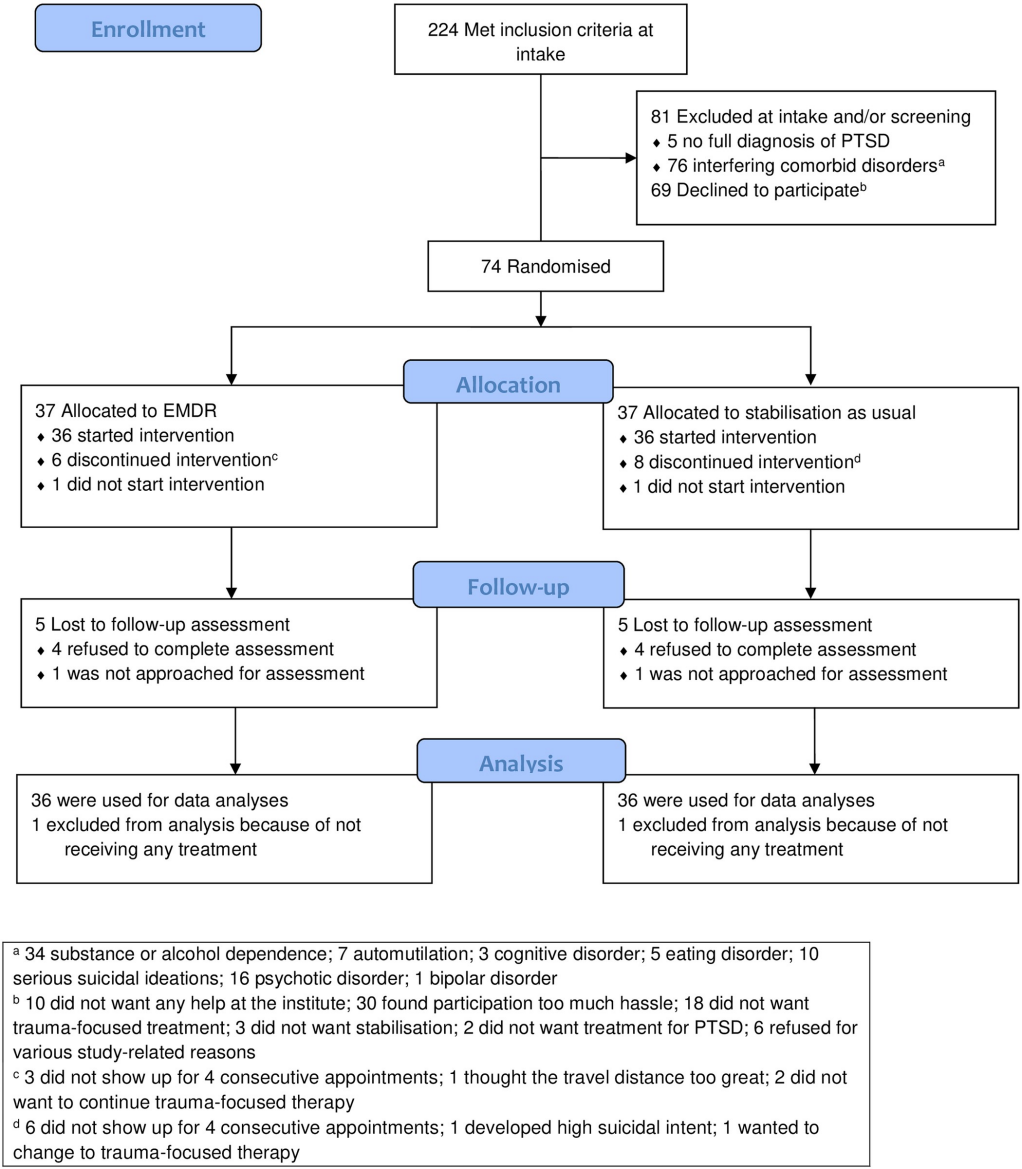
CONSORT 2010 flow diagram.

For the current study aims, the dataset was used in two ways in concordance with the different research questions. First, longitudinal changes were analyzed for which the entire cohort was used and the different treatment arms were disregarded, in line with previous work on the same dataset [29]. Second, by discerning the two treatment arms and comparing outcomes between them, comparable to the main analysis of this trial (Fig 1) [11].

### Participants

Refugees were included who (a) were at least 18 years of age; (b) met criteria for a PTSD diagnosis according to the DSM-IV-TR; and (c) had an indication for individual PTSD treatment. Exclusion criteria were: psychological conditions that required acute care (i.e. suicidal intent or psychotic features) or interfered with the ability to follow trauma-focused treatment (i.e. substance dependence or cognitive disorders).

Seventy-two patients were included in the study, 36 in each arm. All participants had applied for asylum in the Netherlands: one person was undocumented, 12 were in the asylum procedure, 13 had a temporary residency permit, 30 had an indefinite residency permit and 16 had Dutch nationality. Participants were mostly men (n = 52, 72%), had a mean age of 41 years, and originated from 24 different countries (Iraq n = 17, 24%; Afghanistan n = 15, 21%; Iran n = 6, 8%; Bosnia-Herzegovina n = 4, 6%; Sudan n = 4, 6%; several other countries n = 26, 35%). In both groups 20 participants were treated with the help from an interpreter.

### Procedure

The trial was conducted at an outpatient mental health clinic in the Netherlands, between September 2009 and August 2012. Patients who met the inclusion criteria and provided written informed consent were administered a MINI International Neuropsychiatric Interview [26] to verify inclusion and exclusion criteria. Those who were eligible were then randomly assigned to 12 hours (9 sessions) of EMDR therapy or 12 hours (12 sessions) of stabilization. This resulted in an equal number of treatment hours and maximum ecological validity towards the original session duration of each treatment intervention. Measurements and treatments were offered in Dutch with help from (face-to-face or telephone) interpreters when necessary. The research concerned a single-blind study: researchers conducting the measurements were blind to treatment condition, and participants were requested to withhold any details about their treatment during administration. Some of the authors were involved in the data collection (JJtH and DM), but all other authors had no access to information that could identify individual participants during or after data collection. JJtH and DM were not involved in the data analysis.

### Interventions

Therapist manuals containing information on the study were used by the study therapists.

The EMDR condition was performed by clinicians who had successfully completed accredited advanced EMDR courses. The first three treatment sessions (60 minutes each) were focused on treatment planning and preparation. This included practical coordination, psychoeducation on PTSD and EMDR, exploring the patients’ explanatory model, making a timeline of traumatic experiences and symptoms, and selecting memories for desensitization. Subsequently, six session (90 minutes each) were performed using the Dutch version of the EMDR protocol [27], without adding any stabilizing interventions.

The stabilization condition was performed by clinicians who were all experienced in conducting stabilizing interventions. The intervention consisted of twelve sessions (60 minutes each) matching the patient’s needs and without performing trauma exposure. This resulted in interventions focused on increasing safety, control over symptoms, and increasing psychosocial skills (e.g., emotion regulation, stress management). To optimize ecological validity, non-structured stabilization was chosen over a protocolled form of stabilization treatment.

### Meassures

#### PTSD

The Clinician-Administered PTSD Scale for DSM-IV (CAPS-IV) [28] was used to measure PTSD diagnosis and severity. The interview measures frequency and intensity for all 17 DSM-IV PTSD symptoms in the past week and has a total of 30 items. Symptoms are rated on a 4-point Likert -scale, resulting in a maximum score of 8 per symptom. Higher scores indicate more PTSD symptom severity and the total score ranges from 0 to 136 [29]. The CAPS has strong psychometric properties and is considered the ‘golden standard’ for measuring PTSD [30]. The transcultural applicability of this instrument is established [31]. Cronbach’s alpha of the scale was .86 in the current study.

#### Coping

Coping was measured using the self-report Cope-Easy questionnaire [32], which is a Dutch adaptation of the COPE questionnaire [33]. The scale includes 32 items rated on a 4-point scale ranging from 1 (*not at all*) to 4 (*a lot*). Participants are asked to rate how they generally react to difficult situations, with higher scores indicating more available coping relative to lower scores. Items are clustered into four subscales, ranging from 1 to 4, that match coping styles: problem-focused (an average of the mean scores of suppression, active coping, and planning), support-seeking (an average of the mean scores of venting of emotions, emotional social support and instrumental social support), emotion-focused coping (an average of the mean scores of restraint coping, acceptance, positive reinterpretation, religion, and humor) and avoidance (an average of the mean scores of substance abuse, denial, behavioural disengagement and mental disengagement) [34]. The psychometric properties and transcultural applicability of the scale are sufficient [14, 35].

Cronbach’s alpha for the main coping scales problem-focused coping (.83), emotion-focused coping (.78), and support-seeking (.70) were adequate. Cronbach’s alpha for the avoidance scale was .40, indicating a low level of internal consistency for this particular scale. Examination of the subscales underlying the avoidance scale showed that internal consistency of the denial (.74) and mental disengagement (.66) subscales was adequate. Internal consistency of the substance abuse (.28) and behavioral disengagement (.40) subscales, however, was low. The substance abuse subscale consists of four items representing abuse of four different substances (i.e., alcohol, medication, cigarettes, and painkillers) which are conceptually independent (e.g., abuse of alcohol is not necessarily informative for abuse of medication). Hence, the highest score on the four subscale abuse items was deemed to be a more appropriate operationalization of the substance abuse subscale compared to the original operationalization of the mean across all four items. Additionally, we removed behavioral avoidance because of its low reliability (.40) and its low inter-item correlation with the other categories. Its relatively poor performance is in line with previous studies [32]. This make sense when considering the behavioral avoidance questions (e.g., “I went to movies or watched TV to think about it less”), which require an action tendency that contradicts questions from the other categories such as mental disengagement (e.g. “I reduced the amount of effort I was putting into solving the problem”). The adaptions resulted in a Cronbach’s alpha of .56.

### Statistical analyses

The sample size needed for analyses of the primary outcome of the RCT (PTSD) as reported in the original paper, [11] was calculated with the power analysis program G*Power version 2 for Windows [36]. Power calculations were based on the outcomes of a pilot study [37], which resulted in a medium effect size between EMDR and stabilisation on the primary outcome (PTSD). For the RCT, a sample size of 36 patients per condition was needed (using a power of 0.80, a two-sided significance level of 0.05 and three repeated measures) to detect a medium between-treatment effect size at T3. The sample size was based on analyzing group differences as performed in the ANOVA. Nonetheless the sample size was also deemed suitable for the regression analysis and t-test [38].

All analyses were performed with SPSS version 27 using the intent-to-treat strategy. The amount of missing data varied between (sub-) scales and time-points, with no missing data on the CAPS-IV on T1 and 19% for support seeking on T1. These missings were considered at random. The Markov Chain Monte Carlo (MCMC) method was used to impute 50 datasets with 25 iterations. To check for inconsistencies between the original and imputed sample, all analyses were performed on the imputed and original dataset. Prior to the analyses all assumptions were tested and taken into consideration.

CAPS-IV difference scores were calculated by subtracting T1 CAPS-IV scores from T3 CAPS-IV scores (T3-T1). For this part of the data analysis the different treatment arms were disregarded. ‘Responders’ were defined as a CAPS-IV reduction of at least 10 points, non-responders or deteriorating responders were classified as participants with a CAPS-IV increase or a reduction of 9 points or less [39] and labelled ‘non-responders’. Two-tailed independent t-tests were used to test if treatment responders, in terms of reduction in PTSD symptom severity, differed with regard to the four main coping styles at baseline from non-responders. A Bonferroni correction was applied to avoid the risk for Type 1 error due to multiple testing [40]. Therefore, significance level was set at .05/5 = .01. In addition, Cohen’s d effect sizes were calculated, with the following interpretation: small = .2, medium = .5, large = .8 [41, 42]. To check for differences between the original and adapted avoidance scale, results were run for both versions.

Additionally, we tested if baseline coping style levels were associated with change in PTSD symptom severity, using multiple regression analysis. In this part, the full cohort was analyzed indiscriminate of treatment condition. The dependent variable treatment outcome was defined as the CAPS-IV difference between T1 and T3 (T3-T1). This is a suitable approach, when one controls for the potential confounding effects of baseline scores of the change variable [43]. The independent variables, pre-treatment problem-focused, support-seeking, and emotion-focused coping, and the adapted version of avoidant coping were regressed on treatment outcome. Due to the limited sample size, T1 CAPS-IV scores were not directly added to the regression model as a covariate. When the difference score was found to be significantly predicted by one or more of the independent variables, T1 CAPS-IV scores could subsequently be added to the model to check for possible confounding. Prior to this analysis, Pearson correlations were calculated to evaluate the univariate relations between treatment outcome and the different coping styles. Due to the high intercorrelation between the original avoidant coping and the adapted version of avoidant coping, it was impossible to simultaneously enter both in the analysis and solely the adapted version of the avoidance scale was included. To check whether the original and adapted avoidance scale yielded different results, two multiple regression models were tested: the first containing the adapted and the second containing the original avoidance scale. Since the model with the original avoidance scale was added as a check, only inconsistencies, if present, are reported. Variance Inflation Factors were considered to ensure the independency of the different independent variables.

To test whether coping styles change during treatment and whether changes differ between treatment conditions, general linear models (GLM) with the three measurement occasions T1, T2 and T3 (within-subjects factor) and the treatment conditions EMDR and stabilization (between-subjects factor) were used. Hence, this part discriminated between the two arms, in accordance with the design of the original RCT. For each of the coping style scales a separate GLM was tested. Significance of the main effect of the within-subject factor was tested which is indicative of a significant change in coping style during treatment. A Bonferroni correction was applied to avoid the risk for Type 1 error due to multiple testing [40]. Therefore, significance level was set at .05/5 = .01. Effect sizes (η_p_^2^), were added for this step, with the following interpretation: small = .01, medium = .06, large > .14 [42]. Significance of the interaction effect between the within- and between-subjects factors was tested which is indicative of a significant difference in the magnitude of change in the coping style between the treatment conditions. When the assumption of sphericity was violated, the degrees of freedom were corrected for this scale using the Greenhouse-Geisser correction. To check whether the original and adapted avoidance scale yielded different results, analyses were also performed on-the original version of the avoidance scale. If inconsistencies appeared, they were taken into account.

## Results

### Descriptives

Participant characteristics are presented in Table 1. All coping styles were rated between 2 (a little) and 3 (medium) with the highest mean for problem-focused coping.

**Table 1:** Sample descriptives of EMDR versus stabilization and total sample.

Characteristic	EMDR	n	Stabilization	n	Total sample	n
Age in years mean (sd)	43.1 (10.7)	36	39.8 (11.8)	36	41.46 (11.34)	72
Women	17%	6	39%	14	28%	20
Treatment dropout	17%	6	22%	8	19%	14
*CAPS-IV scores*						
T1 mean (sd)	74.67 (18.06)	36	78.25 (18.34)	36	76.46 (18.16)	72
T2 mean (sd)	67.37 (23.15)	32	68.86 (26.93)	29	68.08 (24.82)	61
T3 mean (sd)	69.94 (25.07)	32	69.55 (25.05)	31	69.75 (25.86)	63
*Cope-easy scores*						
Problem-focused coping						
T1 mean (sd)	2.67 (.68)	35	2.85 (.75)	34	2.76 (.71)	69
T2 mean (sd)	2.68 (.63)	29	2.66 (.64)	30	2.67 (.63)	59
T3 mean (sd)	2.53 (.85)	30	2.72 (.63)	30	2.62 (.75)	60
Support-seeking coping						
T1 mean (sd)	2.27 (.57)	35	2.50 (.73)	35	2.39 (.66)	70
T2 mean (sd)	2.43 (.74)	28	2.59 (.81)	30	2.52 (.78)	58
T3 mean (sd)	2.37 (.66)	30	2.46 (.56)	30	2.41 (.61)	60
Emotion-focused coping						
T1 mean (sd)	2.34 (.54)	35	2.61 (.63)	34	2.47 (.60)	69
T2 mean (sd)	2.37 (.55)	29	2.48 (.64)	30	2.42 (.59)	59
T3 mean (sd)	2.50 (.61)	29	2.51 (.58)	30	2.51 (.59)	59
Avoidant coping						
T1 mean (sd)	2.37 (.51)	35	2.35 (.40)	34	2.36 (.46)	69
T2 mean (sd)	2.10 (.46)	30	2.32 (.56)	29	2.21 (.52)	59
T3 mean (sd)	2.35 (.49)	29	2.39 (.50)	30	2.37 (.49)	59
Avoidant coping adapted						
T1 mean (sd)	2.65 (.79)	35	2.59 (.53)	34	2.62 (.67)	69
T2 mean (sd)	2.29 (.59)	30	2.55 (.79)	30	2.42 (.71)	60
T3 mean (sd)	2.52 (.73)	29	2.56 (.72)	30	2.54 (.72)	59

*Note*. T1 = baseline; T2 = post-treatment; T3 = follow-up; CAPS-IV = The Clinician-Administered PTSD Scale for DSM-IV.

### Comparison of baseline coping scores between responders and non-responders

Results of the t-tests are reported in Table 2. There were no significant differences between treatment responders and non-responders with regard to any of the coping styles at baseline. These findings were consistent between the original and imputed sample. In both samples effect sizes for the differences in baseline coping styles between responders and non-responders appeared to be small (Cohen’s d < .50).

**Table 2:** T-test on the predictive value of coping styles for PTSD treatment response between responders and non-responders.

Variable	Non-responders			Responders			df	t	*p*	*d*
	N	M	SD	N	M	SD				
*Original sample*										
Problem-focused	37	2.73	.67	23	2.80	.76	58	0.40	.69	-0.08
Support-seeking	37	2.46	.59	24	2.37	.74	59	-0.56	.58	0.11
Emotion-focused	37	2.51	.63	23	2.51	.56	58	0.01	.99	0
Avoidant	37	2.45	.43	23	2.24	.46	58	-1.82	.07	0.32
Avoidant-adapted	37	2.75	.62	23	2.51	.72	58	-1.40	.17	0.29
*Imputed sample* *										
Problem-focused	41.3	2.71	.66	30.7	2.82	.75	2893	0.62	.53	-0.13
Support-seeking	41.3	2.42	.61	30.7	2.35	.71	1457	-0.40	.69	0.09
Emotion-focused	41.3	2.48	.61	30.7	2.45	.56	1114	-0.20	.84	0.04
Avoidant	41.3	2.46	.42	30.7	2.21	.45	3100	-2.19	.03	0.38
Avoidant adapted	41.3	2.75	.60	30.7	2.45	.70	5436	-1.80	.07	0.37

*Note*. *d* = effect size Cohens’ d.

*The fraction is a consequence of the missing data in the discriminator variable. Based on changes in CAPS-scores, 37 non-responders and 26 responders were identified, while 9 values were missing, in the original dataset. This parameter (changes in CAPS-scores) was used as a discriminator in the t-test. The 9 missing values in this column were filled in during imputation (i.e. people are assigned to one of the two categories during imputation). Their allocation varied per imputed data set. The total always amounts to 72 cases, but the average distribution over responder/non-responder differs resulting into fractions.

### Associations between baseline coping styles and change in PTSD symptom severity

Pearson correlations are presented in Table 3. Weak and non-significant relations were found between the coping styles and PTSD symptom severity. Significant positive correlations were found between support-seeking and problem focused coping, and emotion-focused and problem-focused coping, and emotion-focused and support-seeking coping. This indicates that higher levels in one of these coping style scales were associated with higher levels in the other coping style scale. As expected, a very strong and significant correlation was found between the original and adapted avoidant coping scale, indicating that both concepts highly overlapped.

**Table 3:** Pearson correlation matrix on the relations between change in PTSD symptom severity and coping styles.

Variable	1	2	3	4	5	6
*Original sample*						
1.PTSD change	-					
2. Problem-focused coping	.06	-				
3. Support-seeking coping	.09	.29*	-			
4. Emotion-focused coping	.02	.54**	.49**	-		
5. Avoidant coping	.10	.09	.05	.26*		
6. Avoidant coping adapted	.05	-.02	.01	.05	86**	
*Imputed sample*						
1. PTSD change	-					
2. Problem-focused coping	.001					
3. Support- seeking coping	.08	.29*				
4. Emotion-focused coping	.06	.53**	.49**			
5. Avoidant coping	.18	.08	.04	.26*		
6. Avoidant coping adapted	.14	-.03	.01	.05	.85**	

*Note*. PTSD change = CAPS-IV T3 –CAPS-IV T1;

* *p* < .05 (2-tailed);

** *p* < .001 (2-tailed)

Table 4 summarizes the findings of the multiple regression model. Overall, baseline coping styles contributed minimally to the variation (original dataset: R^2^ = .016; imputed dataset: R^2^ = .034) in PTSD symptom reduction (original dataset: *F*(4, 55) = .22, *p* = .93; imputed dataset: *F*(4, 63.6981) = .40, *p* = .81). None of the individual baseline coping styles were significantly associated with change in PTSD symptom severity. VIF values indicated that the overlap between the coping scales was acceptable. Results were similar in the original as well as the imputed sample, and the model including the original coping scale yielded similar results.

**Table 4:** Multiple regression analyses on the predictive value of coping styles for PTSD treatment response.

Predictor	*B*	*SE B*	β	*t*	*p*	VIF*	R2
*Original Sample*							
Constant	-18.46	19.87		-0.93	.*36*		.016
Problem-focused	2.11	5.31	.06	0.40	.*69*	1.47	
Support-seeking	3.96	5.35	.11	0.74	.*46*	1.28	
Emotion-focused	-2.64	6.79	-.07	-0.39	.*70*	1.75	
Avoidant-adapated	1.83	4.64	.05	0.39	.*70*	1.00	
*Imputed sample*							
Constant	-26.68	20.11		-1.33	.*19*	NA	.034
Problem-focused	-1.33	5.28	-.04	-0.25	.*80*	1.46	
Support-seeking	2.69	5.47	.07	0.49	.*62*	1.37	
Emotion-focused	1.70	6.88	.04	0.25	.*80*	1.76	
Avoidant adapated	4.91	4.68	.13	1.05	.*29*	1.01	

*Note*. Original sample N = 60; Imputed sample N = 72.

*B* = Unstandardized regression coefficients; β = standardized regression coefficients; VIF = Variance Inflation Factor

* = Maximum Variance Inflation Factor based on averaged VIF’s of all imputed datasets for outcomes related to imputed sample.

### Changes in coping style scores during treatment

Results of the mixed method ANOVA are listed in Table 5.

**Table 5:** Mixed method ANOVA on the impact of treatment on coping styles.

Predictor	F	*df*, *error df*	*p*	η_p_^2^
*Original Sample*				
*Within-subjects factor*				
Problem-focused	0.21	2.00, 102.00	.*81*	.*004*
Support-seeking	1.43	2.00, 102.00	.*24*	.*027*
Emotion-focused	0.56	2.00, 102.00	.*57*	.*011*
Avoidant	2.09	2.00, 100.00	.*13*	.*040*
Avoidant-adapted^1^	1.61	1.76, 89.54	.*21*	.*031*
Within*Between subjects interaction				
Problem-focused*condition	0.70	2.00, 102.00	.*50*	
Support-seeking*condition	0.55	2.00, 102.00	.*58*	
Emotion-focused*condition	1.01	2.00, 102.00	.*37*	
Avoidant*condition	1.28	2.00, 100.00	.*28*	
Avoidant-adapted*condition	1.21	1.76, 89.54	.*30*	
*Imputed Sample*				
*Within-subjects factor*				
Problem-focused	1.24	2.00, 2849.77	.*29*	.*017*
Support-seeking	0.47	2.00, 1189.80	.*62*	.*007*
Emotion-focused	0.70	2.00, 1204.34	.*50*	.*010*
Avoidant	3.07	2.00, 1800.40	.*05*	.*042*
Avoidant-adapted	1.91	2.00, 4226.22	.*15*	.*027*
Within*Between subjects interaction				
Problem-focused *condition	0.52	2.00, 1850.49	.*60*	
Support-seeking*condition	0.22	2.00, 2981.25	.*80*	
Emotion-focused*condition	0.94	2.00, 2581.53	.*39*	
Avoidant*condition	1.05	2.00, 8817.51	.*35*	
Avoidant-adapted*condition	0.74	2.00, 4548.48	.*48*	

*Note*. Original sample size = Problem-focused: EMDR n = 27; Stabilization n = 26;

Emotion-focused: EMDR n = 26; Stabilization n = 27; Support-seeking: EMDR n = 26; Stabilization n = 27; Avoidant: EMDR n = 26; Stabilization n = 26; Avoidance-adapted: EMDR n = 26; Stabilization n = 27; Imputed sample size for all coping styles: EMDR n = 36; Stabilization n = 36.

^1^ The assumption of sphericity was violated for the adapted avoidance scale in the original dataset, therefore the degrees of freedom were corrected for this scale using the Greenhouse-Geisser correction.

None of the coping styles changed significantly between T1, T2 and T3, and effect sizes were small (η_p_^2^ < .06), and for support-seeking in the imputed sample and problem-focused in the original sample even very small (η_p_^2^ < .01). There were also no significant differences between the treatment conditions in the amount of change in any of the coping styles between T1, T2, and T3. In line with the lack of significant findings, Table 1 reveals that there were small differences on mean coping style scores between T1, T2 and T3 in each treatment condition and for the whole sample.

## Discussion

This study examined 1) the role of coping styles in PTSD reduction after treatment, and 2) the role of PTSD treatment in changes in coping styles during EMDR versus stabilization in treatment-seeking refugees. Results show that treatment-seeking refugees with PTSD employ a broad range of coping styles (problem-focused, emotion-focused, support-seeking and avoidant), higher than reported during a previous measurement among a comparable sample [14]. However, our hypotheses were only partly confirmed.

### No predictive value of coping styles for PTSD treatment response

We expected higher baseline avoidant coping to be related to more treatment non-response and less PTSD reduction. Additionally we expected higher baseline problem-focused coping to be related to more PTSD reductions and treatment response. For emotion-focused and support-seeking coping styles we expected no relation to PTSD treatment outcomes. None of the baseline coping styles (problem-focused, emotion-focused, support-seeking and avoidant) were significantly related to changes in PTSD symptoms during treatment. Our findings confirmed the hypotheses on emotion-focused and support-seeking coping but confuted the expectations regarding avoidant and problem-focused coping. Since avoidance is a central symptom of PTSD, it was likely that avoidant coping would be related to PTSD symptoms. Moreover, this relation was validated cross-sectionally among other refugee samples [44, 45], one of which from the same institute as the current study [14]. The latter study also fueled our expectations on problem-focused coping for PTSD symptom reductions. Our inconsecutive findings show that baseline coping styles are no prerequisite for PTSD changes after treatment, and require further explanation.

In the current study, coping was measured as a personal, instead of a contextual, resource. Earlier longitudinal investigations confirm the limited relevance of comparable individual factors (e.g., self-efficacy and social engagement) [46] for subsequent PTSD among refugees. In line with this, our findings question the potency of personal resources for altering PTSD in this group [47]. The buffering effects of individual resilience factors, like coping [17], may reach a ceiling effect, given the challenging conditions that refugees face, and consequently lose their protective value [48]. Earlier research suggested that social mechanisms, like post-migration stressors, can undermine the potential effects of coping among refugees [49]. Hence, strengthening patients prior to their PTSD treatment, via improvements in individual coping styles, does not seem helpful based on our data.

### No changes in coping styles after treatment

Contrary to our expectations, coping styles did not change significantly after treatment, nor between treatment conditions. Although EMDR therapy and stabilization target different change mechanisms [11], neither had any impact on coping styles in this study. Hence, increasing coping styles via time-limited EMDR or stabilization treatment seems unfeasible based on our findings. This is a saillant finding, since addressing coping styles is seen as a common factor of several trauma-focused therapies [21].

A potential explanation is that coping can be seen as a persistent trait, instead of more volatile state characteristic [50]. Since refugees are challenged by postmigration stressors [51], they have had the unfortunate opportunity to become aware of their coping styles in many situations. This could result in response tendencies with an overarching self-knowledge based on profound coping experiences. Therefore the questionnaire may have evoked responses that are based on persistent tendencies, although it was developed to capture both situational coping responses and general coping tendencies [33]. Additionally, the number of treatment sessions can impact treatment effects among refugees [52], which can explain the relatively limited impact of short interventions. In light of our findings, expectations on the flexibility of general coping styles after short-term treatments for refugees should be tempered.

Another explanation for the absent changes in coping styles is the refugee context. Their daily life circumstances often contain challenges beyond individuals’ control, which can impair coping skills in the long run [53]. This can counterbalance positive changes that might occur in treatment. This is especially relevant since the majority of participants showed no reliable improvement in PTSD after treatment [11] and few other predictors for PTSD change were significant [29] in previous work on the same dataset. Additionally, meta-analytic findings show that the majority of treated refugees remain symptomatic after PTSD interventions [10]. The difficult living conditions that refugees face negatively impact their PTSD levels [46], and may have comparable impact on coping styles resulting in diminished treatment effects on this outcome.

### Strengths and limitations

The current study has several limitations. First, the COPE-easy brought some concerns. Because the COPE-easy is a self-report questionnaire there is a risk of response bias [54]. The cultural representativeness of the COPE-easy is another relevant issue. The study sample is culturally heterogeneous, with participants originating from 24 different countries, while coping styles can differ between cultural groups [55]. Participants may employ culture-specific coping styles which are not included in the scale, but matter for PTSD symptoms or treatment response. Second, the RCT was performed in a specialized treatment setting offering care to a highly complex refugee population. Therefore it cannot be ruled out that current findings should be assigned to characteristics of the study which limit the generalizability to less complex refugee patients. Third, the sample size was based on PTSD as a primary outcome during a pilot study instead of changes in coping styles. Therefore the sample size may not be sufficient for the analysis applied in the current paper. However, the absolute scores indicate that changes in coping styles after treatment are minimal, and relations between baseline coping style scores and PTSD changes are rather weak. Nonetheless, we cannot rule out that a larger sample would have displayed different results. Last, the data collection was performed before the CAPS-5 was released, which undermines the generalizability to the current understanding of PTSD.

Notwithstanding these limitations, several strengths are worth mentioning. Associations between coping styles and PTSD over time were examined, which is an unsaturated domain in scientific literature on refugee wellbeing [56]. Our findings allow us to draw conclusions on general PTSD change, but also on response categories (responders versus non-responders) which increases the clinical relevance. Additionally, the RCT design allowed us to study the different impact of EMDR and stabilization on coping styles. Consequently, our conclusions may less likely be assigned to a specific type of treatment. This is especially relevant when taking the difficulties in conducting research among refugee populations into account [57], which unfortunately limits the amount of RCTs performed among refugees. Lastly, a profound and well-considered effort was made to improve the avoidance scale, in line with earlier noted challenges [32]. Herewith the current study contributes to the psychometric strength of the COPE-easy.

### Recommendations for future research

Based on the current study, serveral recommendations can be made for future work. Approaching coping as a contextual instead of an individual factor would be suitable for the study population. Future studies should explore if improving community and social factors, that transcend individual resources and increase the ability to cope with daily life, can accelerate the course of PTSD treatment. For example, considering the impact of addressing discrimination or improving language skills is useful, since these factors are related to refugees’ mental health [58].

To gain knowledge on the specific role of treatment for coping styles, our findings might be compared with the natural course of coping styles among the research population. Longitudinal studies on the trajectories of coping styles among traumatized refugees in a naturalistic setting could clarify how coping styles develop without treatment. Additionally, to find a definite answer on the impact of treatment on changes in coping, it is recommended to evaluate the effect on coping styles of treatments with a higher frequency [59] or a higher number of sessions [60] than the current study protocol trial allowed for.

The adapted version of the avoidant coping subschale should be retested among other samples to decide if our operationalization is an improvement. Also, scoring criteria for the COPE-easy should be developed, to add meaning to outcomes of this instrument. Additionaly, future studies should consider the cultural orientations of participants on coping styles. Explorative research is suitable to determine which specific coping styles are relevant for refugees taking on PTSD treatment [61].

## Conclusion and implications

In conclusion, we did not find any evidence that coping styles matter for short-term PTSD treatment followed by refugees at a specialist mental health setting. PTSD changes did not rely on pre-treatment coping styles. Based on our findings, increasing pre-treatment coping styles among refugees receiving short-term therapy, is not recommended for reducing PTSD. Additionally, treatment did not influence coping styles. Hence, improving coping styles via short-term PTSD treatments seems unfeasible for refugees, and should be done by longer or different treatments.

The impact of other operationalizations of coping styles on PTSD should be researched, and other refugee samples should be included, to control the generalizability of our findings. Additionally, the adapted avoidance scale should be retested to control its’ validity. Evaluating the impact on coping styles of treatments with more sessions or higher frequencies is warranted, as well as revealing the natural course of coping styles over time. Future research should meanwhile examine the impact of other change mechanisms for diminishing PTSD symptoms in this highly challenged refugee population.

## Supporting information

S1 Checklist Reporting checklist for randomised trial. (DOCX)
